# Diagnosis and Treatment of Primary and Secondary Lung Cancers

**DOI:** 10.3390/cancers13030448

**Published:** 2021-01-25

**Authors:** Francesco Petrella

**Affiliations:** 1 Department of Thoracic Surgery, IEO European Institute of Oncology IRCCS, 20141 Milan, Italy; francesco.petrella@ieo.it or francesco.petrella@unimi.it; Tel.: +39-0257489362; Fax: +39-0294379218; 2 Department of Oncology and Hemato-Oncology, Università degli Studi di Milano, 20141 Milan, Italy

Primary and secondary lung cancers are the most common clinical conditions that thoracic surgeons have to deal with: primary lung cancer, in fact, is one of the most frequently diagnosed cancers and is the leading cause of cancer-related death worldwide [1]. On the other hand, the lung is the second most common site of metastases and pulmonary metastasectomy is the most frequent surgical resection undertaken by thoracic surgeons [2]. It has been estimated that more than 1600 deaths per day—due to cancers—will be recorded in the US in 2021, the vast majority of deaths being from tumors of the lung, prostate, and colorectum in male patients and tumors of the lung, breast, and colorectum in female patients [1]. Locally advanced non-small cell lung cancer (NSCLC) is a complex disease and the best therapeutic option—ranging from surgery alone to induction treatments or definitive chemo-radiotherapy—remains unclear [3]. We have already reported that “rescue” lung resection is a feasible therapeutic option to maximize overall survival in the case of recurrent or persistent lung tumors after chemo-radiotherapy, in particular in the case of radical resection. However, although feasible, extended lung resection after chemo-radiotherapy is a very challenging procedure, whose morbidity and mortality are not negligible, thus meaning patients should be carefully selected [3]. When performing extended lung resection, in particular right pneumonectomy, the risk of postoperative bronchopleural fistula should always be taken into consideration—we have recently proposed a new technique for post-operative bronchopleural fistula treatment by using autologous mesenchymal stromal cells [4]—however, while small-caliber bronchial dehiscence can be effectively treated by this minimally invasive approach, major bronchial disruption still represents a life-threatening postoperative complication and one of the most feared clinical conditions by thoracic surgeons. An appropriate patient selection should rely both on oncologic staging [5] and functional assessment [6]; moreover, a strict follow up strategy should be considered in order to achieve effective treatment of potential short or long-term recurrences [5]. Similarly, predicting tools for postoperative complications needs to be implemented, in order to correctly identify the groups of patients who most benefit from the proposed treatments and to exclude those who may end up with more damage than advantages [7]. Pulmonary metastasectomy is performed to resect pulmonary metastases from extrapulmonary primary tumors with the aim of radically removing all pulmonary nodules detected by preoperative staging imaging procedures and by intraoperative surgical palpation [2].

Pulmonary metastasectomy has gradually become a frequently performed procedure among thoracic surgeons, particularly after the publication of the encouraging results of the International Registry of Lung Metastases and several other retrospective studies concerning pulmonary metastasectomies [8]. More recently, several questions regarding the real efficacy and need for pulmonary metastasectomy, in cases of multiple lesions, have emerged [9,10]; on the contrary, clarifying answers were expected from the pulmonary metastasectomy in the colorectal cancer (PulMiCC) trial, the first randomized trial comparing pulmonary metastasectomy with active monitoring for lung metastases in patients successfully treated for colorectal cancer. Unfortunately, due to recruitment problems, the PulMiCC trial closed early and the Authors were not able to reach the researched statistical endpoints and finally answer the question regarding the efficacy of lung metastasectomy [11]. Nevertheless, analyzing the results from the only 65 randomized patients, the researchers observed a 5-year survival of 38% of treated patients versus 29% of the Pul-MiCC Control patients with untreated colorectal lung [11]. In conclusion, although the survival rate appeared higher than expected in the control groups, the partial results of this suspended trial should be considered, in my opinion, as further support for the local treatment of pulmonary metastases. Finally, non-epithelial pulmonary metastases should be taken into consideration as potential targets for pulmonary metastasectomy, in particular from osteosarcoma and soft tissue sarcomas [12]. In fact, pulmonary metastasectomy is the standard treatment in patients suffering from lung metastases from bone or soft tissue sarcoma, following primary chemotherapy, although radiofrequency ablation has recently emerged as an effective therapeutic alternative [13].
